# The Role of Phosphorylation and Acylation in the Regulation of Drug Resistance in *Mycobacterium tuberculosis*

**DOI:** 10.3390/biomedicines10102592

**Published:** 2022-10-15

**Authors:** Manluan Sun, Sai Ge, Zhaoyang Li

**Affiliations:** 1 School of Medicine, Shanxi Datong University, Datong 037009, China; 2 Institute of Carbon Materials Science, Shanxi Datong University, Datong 037009, China; 3 Center of Academic Journal, Shanxi Datong University, Datong 037009, China; 4 Laboratory of Molecular Biology, National Institute of Diabetes and Digestive and Kidney Diseases, National Institutes of Health, Bethesda, MD 20892, USA

**Keywords:** *Mycobacterium tuberculosis*, acylation, phosphorylation, post-translational modification, drug-resistance

## Abstract

Tuberculosis is a chronic and lethal infectious disease caused by *Mycobacterium tuberculosis*. In previous decades, most studies in this area focused on the pathogenesis and drug targets for disease treatments. However, the emergence of drug-resistant strains has increased the difficulty of clinical trials over time. Now, more post-translational modified proteins in *Mycobacterium tuberculosis* have been discovered. Evidence suggests that these proteins have the ability to influence tuberculosis drug resistance. Hence, this paper systematically summarizes updated research on the impacts of protein acylation and phosphorylation on the acquisition of drug resistance in *Mycobacterium tuberculosis* through acylation and phosphorylation protein regulating processes. This provides us with a better understanding of the mechanism of antituberculosis drugs and may contribute to a reduction the harm that tuberculosis brings to society, as well as aiding in the discovery of new drug targets and therapeutic regimen adjustments in the future.

## 1. Introduction

Tuberculosis (TB) is a chronic and fatal disease caused by *Mycobacterium tuberculosis* (MTB). According to the World Health Organization (WHO), drug-resistant TB can be classified into five types. Single-drug-resistant TB is only resistant to one of the first-line anti-TB drugs. Poly-drug-resistant TB is resistant to more than one front-line anti-TB drug other than isoniazid (INH) and rifampicin (RIF). Multidrug-resistant (MDR) TB is resistant to both INH and RIF at least. Pan-drug-resistant TB is resistant not only to INH, RIF, and fluoroquinolones (FQs) but also to all second-line anti-TB drug injections. RIF-resistant (RR) TB is regarded as resistant to RIF, whether it is resistant to other drugs or not [1]. Although medicines have been used in clinical trials, the emergence of drug-resistant TB has caused the treatments to become unsatisfactory. Moreover, undergoing 18–30 months of clinical therapy increases the possibility of drug resistance occurring, which makes it even harder for patients to fully recover [2,3]. It is estimated that 9.9 million people were affected by TB worldwide in 2021, and the number of affected people may continuously increase in the foreseeable future [1]. The rising number of affected patients brings a heavy burden to both families and society. Hence, a better understanding of the drug-resistant mechanism associated with MTB will directly contribute to TB prevention and treatment in the future.

Early studies indicate that MTB is naturally resistant to many antibiotics because of its special cell structure and metabolic enzymes [4]. For example, differential gene expression or mutation could lead to changes in drug targets [5,6]. Changes in the MTB membrane permeability or drug intake/efflux pump may decrease the concentration of intracellular antibiotics [7]. However, studies in this area were more focused on the developments of drug resistance or drug targets in MTB [5,6,7]. Post-translational modifications (PTMs) were discovered in about one-third of all MTB proteins. These modified proteins are involved in almost all aspects of intracellular physiological activities, regulating the growth process, metabolism, gene expression, and virulence of MTB [8,9,10,11]. Recently, more and more data have been collected to suggest that PTMs may have impacts on tuberculosis drug resistance [8,9,10,11]. This paper systematically summarizes updated research on the impacts of protein acylation and phosphorylation on the acquisition of drug resistance by MTB through acylated and phosphorylated protein regulating processes.

## 2. Lys Acylation

### 2.1. Acetylation

**Figure 1:**
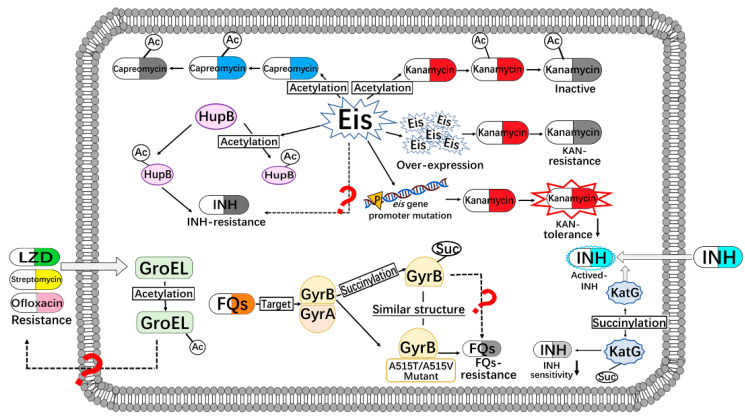
Connections of anti-tuberculosis drugs resistance and protein acylation in MTB. Ac, acetylation; Eis, enhanced intracellular survival protein; FQs, fluoroquinolones; GyrA/B, gyrase subunit A/B protein, INH, isoniazid; KAN, kanamycin; LZD, linezolid; Suc, succinylation.

N-acetylation is a dynamic and reversible post-translational modification. It plays a role in various regulation pathways of MTB. Conventional acetylation is often catalyzed by acetyltransferase (also known as acetylase). Meanwhile, some proteins have been identified as directly acetylated by acetyl donors (such as acetyl co-enzyme A or acetyl phosphate) without enzymes [12]. In contrast, protein deacetylation generally requires deacetylase to catalyze deacetylation [13]. In MTB, the enhanced intracellular survival (Eis) protein is an acetyltransferase that acetylates a second-line anti-TB drug, such as kanamycin (Figure 1). Meanwhile, the overexpression of the *eis* gene in MTB inactivates the antimicrobial function of kanamycin [14]. However, strains obtained from the clinical environment with the *eis* promoter mutation could reduce the kanamycin tolerance [14,15]. In addition, Eis can inactivate capreomycin antibacterial activity by acetylation [16]. On the other hand, a proteome analysis indicated that the Eis protein itself undergoes acetylation modification [17]. Hence, research on Eis mostly focuses on the screening of its inhibitors. However, the following questions remain unclear and should be answered: Does Eis affect the drug action by acetylating proteins in the process of drug tolerance metabolism? Is there a regulating process between the acetylation of Eis itself and aminoglycoside antibiotics resistance?

With the rapid development of proteomics, more and more acetylated proteins in MTB have been identified. Among these acetylated proteins, quite a few were found to be related to drug tolerance. For example, data suggest that PknH, PhoP, MurF, KatG, InhA, RpoB, RpsL, GyrA, and GyrB may have impacts on the resistance of INH, ethambutol (EMB), FQs, kanamycin, and vancomycin [11,18]. However, whether the acetylation is related to the antibiotic resistance is still unclear. In the present study, the putative acetyltransferase Rv2710 in MTB is reported to inactivate INH. The overexpression of Rv2710 in *M. tuberculosis* H37Ra leads to its resistance to INH at MICs [19].

HupB is not only an essential iron regulatory protein for the growth of MTB [20]; it is also a histone-like protein. It can bind to DNA to regulate the expression of the mycobacterial genome [21]. HupB can be regulated by lysine methylation and lysine acetylation, which have a relationship with bacterial phenotypical drug-resistance [22]. The acetylation of HupB at Lys86 can affect the expression of corresponding genes, resulting in INH-resistance in the mycobacterium [22]. In addition, when HupB Lys86 is acetylated by Eis, the binding of HupB to DNA will be affected [23]. However, the relationship between this modification affection and drug-resistant acquisition remains unclear and needs to be further studied.

The expression of the molecular chaperone protein GroEL in MTB is induced under environmental drug pressure, which helps other proteins fold correctly to resist drug pressure. Transcriptome and proteome data show that when MTB is exposed to linezolid (LZD), streptomycin, ofloxacin and/or other antibiotics, the expression of the molecular chaperone GroEL2 increases significantly [24,25]. Meanwhile, data suggest that GroEL2 has multiple acetylated sites, including Lys33, Lys140 and Lys195, but whether those acetylated sites affect drug-resistant acquisition remains unclear and needs to be further studied [17].

### 2.2. Succinylation

Succinylation is a new PTM that was discovered in recent years. It describes the transfer of succinyl group(s) to lysine residues through enzymatic or non-enzymatic catalysis [26]. A succinylated proteome of XDR strains was identified in China for the first time in 2014. InhA, RopB, and GyrA/GyrB were found to be succinylated and related to anti-TB drug targets [27]. Among these proteins, GyrA/GyrB is the target protein of the second line anti-TB drug FQs. Succinylation at Lys523 of GyrB forms a similar spatial structure to the natural mutations A515T or A515V [28], which is related to the tolerance of FQs in MTB. These studies indicate that succinylation is related to FQs resistance through modifying GyrA/GyrB.

Another catalase, KatG, can activate INH and mediate the sensitivity of MTB to INH [29]. The succinylation of KatG at the Lys310 site reduces the sensitivity of MTB to INH, resulting in an increase in INH MIC by 200-fold [11,27,30]. Moreover, the 410th and 688th lysine residues were identified as having the ability to be acetylated, but their significance in drug resistance remains unknown.

## 3. Ser/Thr/Tyr Phosphorylation

Reversible protein phosphorylation plays an important role in the response to external pressure for MTB [31]. In MTB, there are 11 kinds of eukaryotic-like Ser/Thr protein kinases (STPKs, including PknA-PknK), 1 homologous phosphatase, 1 tyrosine kinase and 2 tyrosine phosphatases [32,33,34]. These kinases regulate the phosphorylation of intracellular proteins which, in turn, affects the cell growth and division, gene expression, protein synthesis, pathogenicity and drug resistance of MTB [35,36,37]. All of these processes would also influence the anti-TB drug metabolism in cells.

### 3.1. Phosphorylation of Proteins Related to Cell Wall Synthesis

**Figure 2:**
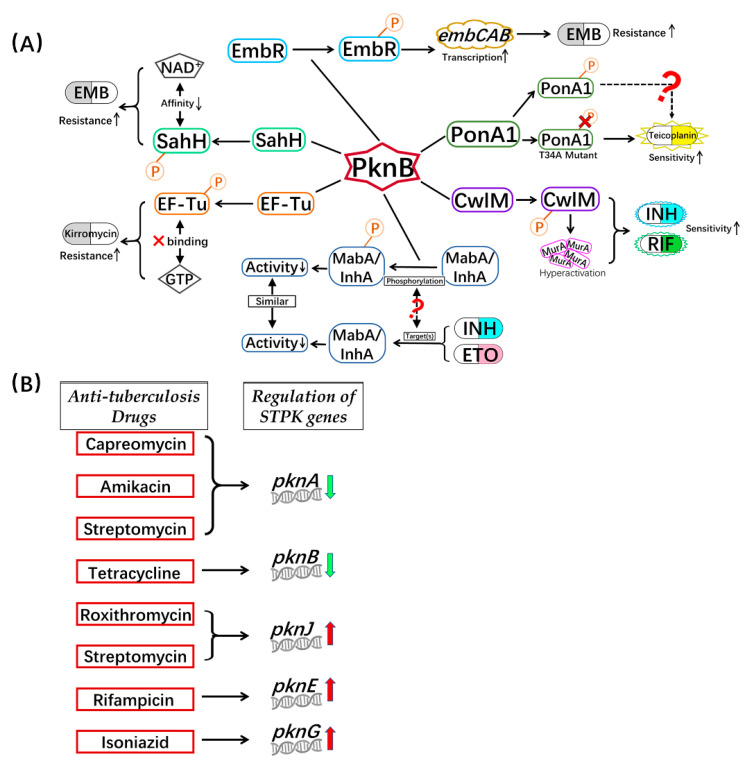
Connections of anti-tuberculosis drugs resistance and protein phosphorylation in MTB. (**A**) PknB mediates drug resistance/tolerance by phosphorylation; (**B**) Regulation of STPK genes after treatment with anti-tuberculosis drugs. EF-Tu, elongation factor Tu; EMB, ethambutol; ETO, ethionamide; GTP, guanosine-5-triphosphate; INH, isoniazid; InhA, 2-*trans*-enoyl-acyl carrier protein reductase; MabA, beta-ketoacyl-acyl carrier protein reductase; MurA, UDP-N-acetylglucosamine enolpyruvyle transferase; NAD+, nicotinamide adenine dinucleotide; P, phosphorylation; RIF, rifampicin; STPK, Ser/Thr protein kinase; SahH, S-adenosyl homocysteine hydrolase.

Similarly to most bacteria, the synthesis of MTB cell walls is a dynamic process, which can profoundly and sensitively respond to environmental stresses. Serine/threonine protein kinase B (PknB) is not only an essential protein involved in regulating MTB cell wall synthesis, it is also a potential anti-tuberculosis drug target (Figure 2A) [38]. Among the substrate proteins phosphorylated by PknB, both CwlM and PonA1 are involved in mycobacterial cell wall formation [39,40,41]. CwlM is a peptidoglycan hydrolase (amidase) that is responsible for the hydrolysis of bacterial cell walls [42]. Together with UDP-N-acetylglucosamine enolpyruvyl transferase (MurA), the enzyme involved in the first step of the synthesis of peptidoglycan, CwlM regulates the biosynthesis of peptidoglycan [42,43,44]. PknB can activate the overexpression of MurA and cell division by phosphorylating CwlM under a rich nutritional environment for MTB growth. Actively dividing mycobacteria are more sensitive to antibiotics such as INH and RIF, compared with cells that are not living in rich nutritional environments [25]. In *Mycobacterium smegmatis*, CwlM is the substrate of Ser/Thr phosphatase PstP. The site-directed mutation (T171E) of PstP, which mimics the phosphorylation of PstP, can affect the antibiotic resistance of bacteria in the growth and lag phases [45]. However, whether the true phosphorylation state in MTB affects drug resistance requires more experimental data.

PonA1 is a penicillin-binding protein that is essential for the maintenance of cell wall biosynthesis and cell shape during MTB growth. PonA1 can perform two activities: transglycosylation and transpeptidation. In MTB and *Mycobacterium smegmatis*, the transpeptidase activity of PonA1 is mostly related to the resistance of antibiotics targeting cell wall synthesis, such as penicillin V and meropenem [46]. The transglycoside activity of PonA1 will be regulated when PonA1 is phosphorylated by PknB at Thr34. This phosphorylation process can affect the balance between these two enzymatic activities of PonA1, which leads to a difference in the antibiotic sensitivity of mycobacteria [41]. Meanwhile, a four-fold increase in the sensitivity to the glycopeptide antibiotic teicoplanin was discovered with a lack of phosphorylation of PonA1 [41]. Therefore, PknB can affect the sensitivity of mycobacteria to antibiotics by regulating the activity of PonA1 through phosphorylation.

**Figure 3:**
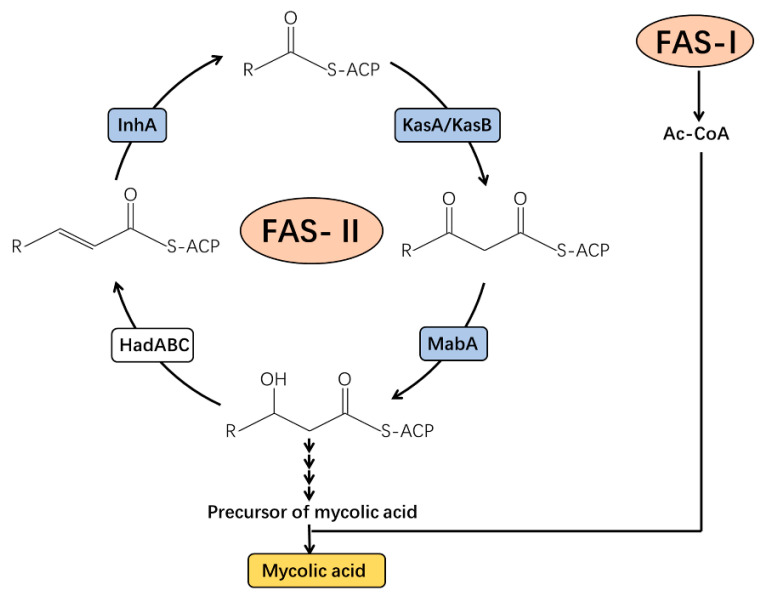
Biosynthesis of mycolic acid in *Mycobacteria tuberculosis.* Ac-CoA, acetyl-coenzyme A; FAS-I, fatty acid synthase typeI; HadABC, beta-hydroxyacyl-ACP dehydratase complex; InhA, 2-*trans*-enoyl-acyl carrier protein reductase; KasA/KasB, beta-ketoacyl-acyl carrier protein synthases A/B; MabA, beta-ketoacyl-acyl carrier protein reductase.

The specific components of the MTB cell wall make its drug resistance mechanisms unique and different from those of other bacteria. In MTB, various proteins have been identified as targets of numerous drugs [37,47,48,49], which participate in the process of mycolic acid biosynthesis (Figure 3). Fatty acid synthase typeII(FAS-II) is a multi-enzyme system that is a target associated with the inhibition of mycolic acid synthesis by drugs in MTB [50]. The active INH Beta-ketoacyl-acyl carrier protein reductase (MabA) and NADH-specific enoyl acyl carrier protein reductase (InhA) are important enzymes associated with FAS-II [51,52]. The activated INH inhibits the InhA protein by binding to its active site which, in turn, hinders mycolic acid synthesis, and disturbs *M. tuberculosis* cell wall biosynthesis [19]. MabA and InhA can be phosphorylated by many kinds of STPKs, including PknB. Three phosphorylated threonine sites in MabA have been identified, of which Thr191is the main phosphorylation residue, while for InhA, Thr66 is the main phosphorylation site [53,54,55]. Phosphorylation of MabA and InhA reduces their enzymatic activities, resulting in a decrease in mycolic acid synthesis and a change in cell wall permeability [56]. Additionally, the *mabA^g609a^* silent mutation results in the upregulation of *inhA*, which may be a novel mechanism associated with INH tolerance [57]. Similarly, MabA and/or InhA are targets of INH and ethionamide (ETO). When MTB is treated with INH/ETO, the expression and function of MabA and/or InhA is inhibited [58]. Why do two different treatment methods have similar effects? Whether the phosphorylation of MabA and InhA is related to the mechanisms associated with INH and ETO drug effects remains to be further studied.

Other important components of the FAS-II system are the beta-ketoacyl carrier protein synthases (KasA and KasB, as shown in Figure 3). They complete the synthesis of the beta-ketoacyl carrier protein (ACP) together [59]. Studies have shown that KasA and KasB participate in the formation of INH resistance by MTB. Through chemical genetic screening, KasB is upregulated by RIF, INH, ETO, vancomycin, and meropenem [60]. KasA and KasB can be phosphorylated by many kinds of STPKs, and their activities decrease after phosphorylation, for example, to MabA and InhA. Meanwhile, mass spectrometry and site-directed mutagenesis show that phosphorylation, or its mimic state of KasB, at the Thr334 or/and Thr336 sites affects the pathogenicity and acid-fast staining of MTB [61]. Therefore, it is speculated that the inhibition of phosphorylation by enzymatic activity regulates the sensitivity of MTB to those drugs.

### 3.2. Phosphorylation of Proteins Related to DNA Metabolism and Transcription

Different from the mechanism by which other bacteria acquire antibiotic resistance through horizontal gene transfer, one of the main mechanisms of drug resistance of MTB is the change in important genes in drug targets and metabolic pathways, including the DNA damage response (DDR) [5,62]. In MTB, LexA is one of the important regulators involved in DDR. RecA, a DNA-dependent ATP enzyme, regulates repair in DDR by interacting with LexA. In the DDR process of MTB, phosphorylation of RecA at Ser207 leads to its inactivation and inhibits its binding process with the LexA repressor. Phosphorylation aggravates DNA damage and is responsible for this inhibition, making MTB resistant to RIF [63].

Data suggest that some phosphorylation transcription factors also participate in the change in MTB drug-resistance. Ethambutol, a first-line anti-TB drug, inhibits the activity of arabinosyltransferase and blocks the synthesis of arabinogalactan. This inhibition regulates the ratio of lipoarabinomannan (LAM) to lipomannan (LM), thereby changing bactericidal effects by affecting the permeability of the cell wall [64]. The EmbR transcription factor regulates the *embCAB* operon, which contains three consecutive genes encoding for arabinosyltransferase. On the other hand, STPKs such as PknA, PknB, and PknH can phosphorylate EmbR to promote its binding with DNA. Hence, the phosphorylation processes increase the transcription of *embCAB* and change the resistance to EMB [65,66].

Ethionamide (ETO), a second-line oral drug for the clinical treatment of tuberculosis, needs to be activated by the monooxygenase EthA to perform intracellular functions [67]. MTB increases its resistance to ETO when the transcription inhibitor EthR binds to the *ethA*-*ethR* area to inhibit the expression of *ethA*, resulting in a reduction in EthA activity [68]. On the other hand, the N-terminal of EthR can be phosphorylated by the kinase PknF at Thr2, Thr3, Ser4 and Ser7. The site-directed mutation of these amino acid residues, which mimics phosphorylation, can reduce the affinity of EthR to DNA, resulting in the inhibition of *ethA* caused by EthR reduction [69]. Therefore, phosphorylation may regulate the ETO resistance of MTB by affecting the relationship between EthR and EthA. However, the specific regulatory mechanisms need more data for confirmation.

### 3.3. Phosphorylation of Proteins Related to Translation

In the process of protein translation, the elongation factor Tu (EF-Tu) delivers aminoacyl-tRNA to the ribosome. Proteomics data show that EF-Tu can be phosphorylated by PknB to reduce the interaction between EF-Tu and GTP, resulting in a reduction in the efficiency of protein translation [70,71]. When EF-Tu is phosphorylated, MTB shows lower sensitivity to kirromycin [71]. Moreover, some researchers have found that the expression of EF-Tu decreases when MTB is treated with INH. Meanwhile, compared with common MTB strains, the expression of EF-Tu from anti-norfloxacin MTB increased twofold. However, the impact of EF-Tu expression and phosphorylation processes remains unclear.

### 3.4. Phosphorylation of Proteins That Participate in Other Biological Processes

MTB regulates the activities of proteins that participate in important metabolism pathways through phosphorylation to adapt to environmental changes. More and more studies are indicating that these enzymes are the targets of anti-TB drugs. Among them, the levels of protein metabolism related homocysteine, S-adenosylmethionine (SAM), and S-adenosylhomocysteine (SAH) are regulated by S-adenosylhomocysteine hydrolase (SahH). The reversible hydrolysis of SAH by SahH needs the assistance of NAD^+^ [72]. It has been shown that the phosphokinase PknB from MTB can phosphorylate SahH at the Thr219, Thr220, and Thr221 residues. These phosphorylated residues are found in the active sites of the enzyme. Phosphorylation processes reduce the affinity of SahH and NAD^+^ and subsequently influence the levels of products in homocysteine metabolism [73]. It has also been reported that SahH is one of the binding proteins associated with the INH-NAD(P) complex. Its phosphorylation processes may affect the interaction between SahH and INH as well as influencing the resistance of MTB to INH [74].

### 3.5. STPKs Involved in Drug Resistance

By analyzing the transcriptome reported in MTB research, we found that the *pknA* gene is downregulated 4.76-, 2.11-, and 2.14-fold after treatment with capreomycin, amikacin and streptomycin, respectively. Meanwhile, the *pknB* gene is downregulated 3.41-fold after treatment with tetracycline. In contrast, following the treatment with roxithromycin and streptomycin, the *pknJ* gene is upregulated 2.14- and 2-fold, respectively. With RIF treatment, the *pknE* gene is upregulated 5.2-fold, and the *pknG* gene is upregulated 2.71-fold following treatment with INH (Figure 2B) [75]. PknG is one of the most important STPKs in MTB. Antibiotic sensitivity was shown to increase in the MTB and *Mycobacterium smegmatis pknG* deletion mutant strains [76]. Instead, after the complementary *pknG* experiment, the drug resistance was restored. However, the drug resistance could not be recovered by complementing with the PknG^K181M^ mutant [77]. Hence, as an important protein kinase in MTB, whether PknG can change the drug resistance by regulating the phosphorylation of its substrate is also one of the research topics for the future.

PknG regulates the central carbon and nitrogen metabolism by regulating the phosphorylation levels of its substrates, which are often related to drug resistance in MTB [78,79,80,81]. Another well-studied substrate of PknG is the GarA protein. GarA can regulate the TCA cycle and nitrogen metabolism negatively by binding to the α-ketoglutarate dehydrogenase complex (KDH). GarA contains a phosphorylation recognition FHA (forkhead-associated) domain [82]. The phosphorylation of GarA reduces its inhibitory effects on KDH [83]. Studies have shown that phosphorylated GarA combines with α-ketoglutarate decarboxylase (KGD), NAD-dependent glutamate deoxy-enzyme (GDH) as well as glutamate synthase (GS) and affects their activities. These enzymes regulated by GarA are involved in the interconversion between NAD^+^ and NADH. Therefore, PknG may affect the intracellular NADH pool by regulating the phosphorylation of GarA. On the other hand, in MTB, intracellular oxidative stress is closely related to drug metabolism and resistance, for example, INH and ETO. Hence, PknG may affect the drug tolerance of MTB by regulating the levels of NADH and free radicals that result in cell death [84,85,86].

Interestingly, acetylation processes have been identified in some STPKs, such as PknD, PknK, PknG, and PknH [11,87]. Moreover, EmbR, which plays an important role in EMB resistance, can be phosphorylated by PknH as well as being modified by acetylation. In addition, those two types of protein modification are also found in Wag31, which is an essential protein and a drug target in MTB [88]. In MDR strains, the expression of Wag31 is upregulated. The site-direct mutant (Q201R) of Wag31 mimics the deacetylated protein, which has been proven to be related to the drug resistance of amino pyrimidine sulfonamide (APYS1) [89]. However, whether acetylation and phosphorylation can double regulate important proteins related to the tolerance of anti-TB drugs is also an interesting research direction for the future.

## 4. Summary and Prospect

With the rapid developments in mass spectrometry, transcriptome and chemotherapy (pharmacomics), more and more post-translational modified proteins of MTB have been identified. Some post-translational modifications originally found only in eukaryotes are gradually appearing in prokaryotes. Acylation and phosphorylation proteins regulate the cell status by becoming involved in almost all aspects of intracellular physiological activities (Table 1). Due to the emergence of drug-resistant tuberculosis, research on its mechanism in MTB has gradually become a hot topic in scientific research.

Although there is a significant body of research on the mechanism by which drug resistance occurs in MTB, research on the roles of acylation and phosphorylation is still in the initial stage. In recent years, acylation and phosphorylation have been identified in large number of proteins in clinical isolates (including standard strains and drug-resistant strains) of MTB [90]. For example, Birhanu et al. found that 953 proteins in MTB are acetylated, and these modified proteins participate in bacterial center metabolism, oxidative stress, environmental pressure and antibiotic tolerance [11]. Xie et al. found that phosphorylated proteins in MTB participate in drug resistance, bacterial virulence, and interactions with the host [31]. Meanwhile, with the extensive use of antibiotics in the treatment of tuberculosis, bacteria evolve to have new and more complex drug resistance mechanisms so that they can quickly perceive drug pressure and regulate their transcription, translation, metabolism, and corresponding enzyme functions. Acylation and phosphorylation, as the most common PTMs, play important regulatory roles in the above processes. However, there are still many unsolved problems. For instance, the previously discussed iron regulatory protein HupB of MTB can be regulated by multiple post-translational modifications, such as acetylation, phosphorylation and methylation. It is known that the acetylation of HupB affects the sensitivity of MTB to INH, but its specific processes are still unclear. At present, research on PTMs is focused on single modifications, but whether modifications regulate and influence each other is also a topic worthy of future discussion.

## Figures and Tables

**Table 1:** Common phosphorylated and/or acylated proteins involved in drug-resistance in MTB.

Protein	Functions	Post-Translational Modifications	Related Antibiotics-Resistance
KatG	catalase peroxidase, INH-resistance	Phosphorylation, acetylation, succinylation	INH
MetK	S-adenosylmethionine synthase, decreased expression after INH treatment	Phosphorylation, acetylation	INH
InhA	Enoyl Acyl carrier protein reductase, inhA transcription change after INH treatment	Phosphorylation, acetylation	INH
MabA	Beta-ketoacyl-acyl carrier protein reductase, participate in INH tolerance together with InhA	Phosphorylation, acetylation	INH
EF-Tu	elongation factor Tu, decreased expression after INH treatment	Phosphorylation, acetylation	INH, kirromycin
KasA and KasB	beta-ketoacyl carrier protein synthases	Phosphorylation	INH, RIF, ETO
HupB	essential regulatory protein, Lys acetylation results in INH resistance	Acetylation, lysine methylation	INH
SahH	S-adenosylhomocysteine hydrolase, INH-NADP complex will be affected after its phosphorylation	Phosphorylation, acetylation	INH
EmbR	transcription factor for embCAB, tolerance to EMB will be changed by its phosphorylation	Phosphorylation, acetylation	EMB
RecA and LexA	phosphorylation of RecA inhibits its binding with LexA repressor	Phosphorylation	RIF
GyrA and GyrB	DNA gyrase	Succinylation, phosphorylation	FQs
GroEL2	molecular chaperone protein, increased expression after LZD, streptomycin, ofloxacin treatment	Phosphorylation, acetylation	LZD, streptomycin, ofloxacin
Eis	enhanced intracellular survival protein	Acetylation	kanamycin, capreomycin
EthA	monooxygenase	Acetylation	ETO
EthR	EthA transcriptional regulator	Phosphorylation	ETO
PonA1	penicillin-binding protein, transglycoside activity of PonA1 will be regulated by phosphorylation	Phosphorylation	penicillin V, meropenem

## Data Availability

Not applicable.
